# Particle implantation combined with chemotherapy for rhabdomyosarcoma of the head and neck: A 8‐year long‐term follow‐up case report

**DOI:** 10.1002/cai2.71

**Published:** 2023-04-20

**Authors:** Sidou He, Shuhang Tian, Na Xu, Jianguo Zhang, Chao Duan, Xiaoli Ma

**Affiliations:** ^1^ Department of Medical Oncology, Pediatric Oncology Center, Beijing Children's Hospital, National Center for Children's Health, Beijing Key Laboratory of Pediatric Hematology Oncology, National Key Clinical Discipline of Pediatric Oncology, Key Laboratory of Major Diseases in Children, Ministry of Education Capital Medical University Beijing China; ^2^ Department of Oral and Maxillofacial Surgery Peking University School and Hospital of Stomatology & National Center of Stomatology & National Clinical Research Center for Oral Diseases & National Engineering Laboratory for Digital and Material Technology of Stomatology & Beijing Key Laboratory of Digital Stomatology Beijing China

**Keywords:** ^125^I seed implantation, chemotherapy, children, head and neck, rhabdomyosarcoma

## Abstract

Rhabdomyosarcomas (RMSs) are highly malignant soft‐tissue sarcomas. Head and neck RMSs often pose unique challenges to treatment because of their closeness to important structures. We here report a rare case of a 1‐year‐old boy with a 1‐month history of right eye swelling and an eye mass. Biopsy of deep tumors in the maxillofacial region supports embryonal RMS. Postoperative positron emission computed tomography showed a 5.0 cm × 4.8 cm × 4.2 cm malignant tumor in the right maxillary region. In accordance with the international RMS study group guideline, the child was diagnosed with IIIa and TNM stage T2bN1M1 embryonal RMS. The child was treated with a combination of chemotherapy and ^125^I seed implantation radiotherapy and eventually achieved partial remission. This case report shows that ^125^I seed implantation is a safe and effective means of delivering radiotherapy to young children with head and neck RMSs. It may be an option for children with RMSs for whom surgery or external radiotherapy is unsuitable.

## Graphical abstract

In this article, children with orbital rhabdomyosarcomas received chemotherapy combined with ^125^I seed implantation radiotherapy without undergoing surgical treatment, resulting in long‐term remission.

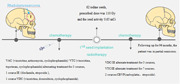

**Abbreviations**
CT: computed tomography
IE: ifosfamide and etoposide
RMS: rhabdomyosarcoma
VAC: vincristine, actinomycin D, cyclophosphamide
VDC: vincristine, doxorubicin D, cyclophosphamide
VTC: vincristine, topotecan, cyclophosphamide

## 1 INTRODUCTION

Rhabdomyosarcomas (RMSs) is the most common malignant tumor of the orbit in children, with about 10% occurring in the orbit [1]. Orbital RMSs typically present with proptosis and ophthalmoplegia [2]. Although head and neck sites of primary RMSs are considered favorable regarding outcome and survival, the complicated anatomy of these sites makes performing surgery and/or administering local radiation therapy difficult for patients aged under 3 years. We here present a 1‐year‐old child with RMS who was treated with chemotherapy combined with ^125^I seed implantation therapy. We will discuss the treatment of this rare case, including the long‐term effects of his local treatment.

## 2 CASE PRESENTATION

A 1‐year‐old boy with a 1‐month history of eye swelling and an eye mass presented to our hospital and was found to have swelling of the right eyelid but no other abnormalities. He was diagnosed as having a stye. During 3 weeks of symptomatic treatment, the right eye mass enlarged to 10 cm × 5 cm × 3 cm. Orbital computed tomography (CT) showed localized bone resorption, destruction of the right maxilla, and a soft tissue shadow. The tumor had invaded the orbit superiorly. A biopsy of the deep maxillofacial tumor was obtained at a local hospital. A pathological examination of the biopsy sample revealed a small cell tumor of the right maxilla. After pathological consultation between three hospitals and immunohistochemistry, the final diagnosis was an EMR. Postoperative positron emission‐CT showed a 5.0 cm × 4.8 cm × 4.2 cm malignant tumor in the right maxillary region. There was extensive invasion and metastases in the right parapharyngeal lymph nodes and sternocleidomastoid muscles bilaterally. No other metastases were found. In accordance with the international RMS study group guideline [2], the child was diagnosed with IIIa and TNM stage T2bN1M1 before commencing treatment.

**Figure 1:**
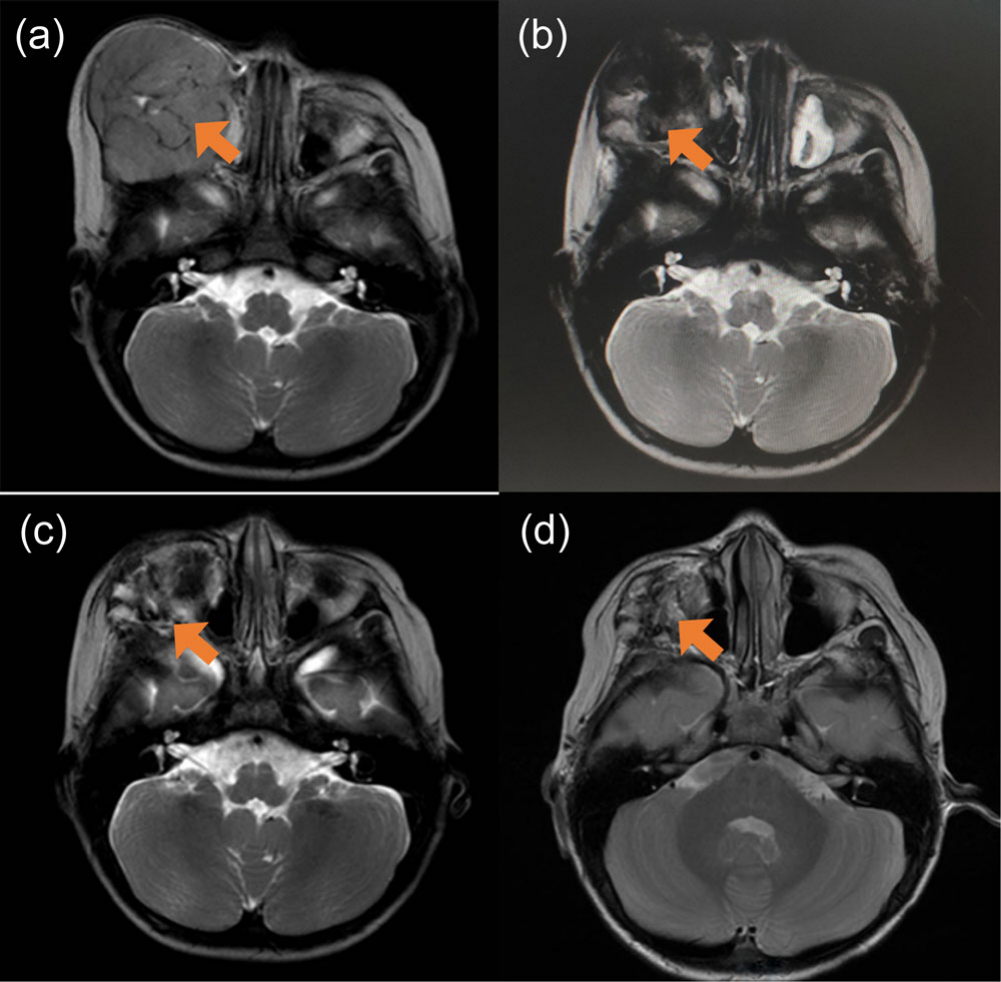
Skull MR showed of patient. (a) After four courses of chemotherapy. (b) After 15 courses of chemotherapy and ^125^I particle implantation. (c) After 17 courses of chemotherapy and ^125^I particle implantation. (d) One year after the end of chemotherapy.

In accordance with the Beijing Children's Hospital (BCH) strategy for medium‐risk RMSs, three courses of vincristine 0.05 mg/kg, actinomycin D 0.045 mg/kg, and cyclophosphamide 2.2 g/m^2^ (VAC)/vincristine 0.05 mg/kg, topotecan 1.5 mg/m^2^, and cyclophosphamide 750 mg/m^2^ (VTC) were administered, followed by one course of ifosfamide 1.8 g/m^2^ and etoposide 100 mg/m^2^ (IE). This did not achieve significant shrinkage of the assessed tumors (Figure 1a). In accordance with the Beijing Children's Hospital protocol for stable, high‐risk disease RMS, the chemotherapy regimen was changed to vincristine 1.5 mg/m^2^, doxorubicin 30 mg/kg, cyclophosphamide 1.2 g/m^2^ (VDC) for one course. This was followed by the implantation of 62 iodine‐125 seeds into the right maxillary tumor under CT guidance. The prescribed dose was 110 Gy and the seed activity was 0.65 mCi. The procedure was performed under general anesthesia, with seeds being implanted at 1 cm intervals. Subsequently, VDC and IE schemes were administered alternately for three courses, followed by VTC and IE for seven courses. This resulted in significant tumor shrinkage to 2.0 cm × 0.6 cm × 0.8 cm (Figure 1b). Next, two courses of carboplatin 200 mg/m^2^ and etoposide 150 mg/m^2^ were administered (Figure 1c). The patient complied with the above chemotherapy protocol and had no serious local adverse reactions. Regular reexamination after chemotherapy, during which no tumor metastasis or recurrence was identified (Figure 1d). After 96 months of follow‐up, our patient continues to be in stable partial remission (PR) according to the criteria for evaluating primary tumor responses in solid tumors.

In addition to assessing the primary tumor, we also conducted a long‐term follow‐up of changes secondary to the tumor and its treatment. The child is now 8.3 years old and growing normally. His height of 131 cm, weight of 28.4 kg, and BMI of 16.5 kg/m^2^ are all within the normal range for his age [3]. He could not open his right eye before treatment but gradually regained the ability to open it after the fifth to sixth course of chemotherapy. His vision is similar in the left and right eyes, but he has mild right‐sided strabismus. His senses of hearing and smell are unaffected. About 1 year after stopping chemotherapy, his right upper incisors turned black. The skin in the right maxillofacial region is darker than elsewhere, but his overall appearance is symmetrical, and he has normal facial movement and development.

## 3 DISCUSSION

RMSs are highly malignant soft tissue sarcoma [4]. Surgery combined with radiotherapy and chemotherapy is currently the main means of achieving the optimal response rate [5]. Our patient had a large tumor without distant metastasis or intracranial invasion. Resection was considered contraindicated because it would have required a difficult surgical procedure and destroyed his facial structure.

External radiotherapy significantly damages local tissues and has serious long‐term adverse effects [6]. ^125^I seed implantation can be performed under the guidance of CT and magnetic resonance imaging. A treatment planning system is used to determine the contour of the target tumor area, eyeball, and visual nerve. Next, the doses to the target area (tumor), eyeball, and optic nerve are calculated, and the dose, activity, quantity, and spatial positioning of ^125^I seeds are adjusted accordingly. Finally, a guided puncture template is constructed by 3D printing technology and used to accurately guide the insertion of the needle and implantation of the ^125^I radioactive seeds, ensuring that the target area is exposed to at least 95% radiation dose while the eyeball. Orbital CT scanning is performed 1–3 days after this procedure to confirm its quality [7]. The greatest advantage of local seed implantation is that the total radiation dose delivered is much lower than that delivered by external radiotherapy. This is of particular importance in children, markedly reducing the probability of local abnormal development [8]. Some children's parents initially refuse ^125^I seed implantation radiotherapy, in which case seed implantation can be performed to treat subsequent local tumor recurrence. ^125^I seed implantation is recommended after three to four cycles of chemotherapy in patients in whom surgery and chemotherapy have achieved partial or complete remission. Seed implantation does not hinder the continuation of chemotherapy and therefore shortens the treatment time compared with external radiotherapy, which necessitates a pause in chemotherapy. Our patient underwent ^125^I seed implantation after completing four cycles of chemotherapy, the effect of which was similar to that of external radiotherapy.

## 4 CONCLUSIONS

Overall, this case report shows that chemotherapy combined with ^125^I seed implantation radiotherapy is a safe and effective treatment approach in young children with head and neck RMS. It may be an option for children with RMS whose tumors cannot be managed with surgery or external radiotherapy.

## AUTHOR CONTRIBUTIONS

**Sidou He**: Writing—original draft (equal). **Shuhang Tian**: Writing—review & editing (equal). **Na Xu**: Data curation (equal); Investigation (equal). **Jianguo Zhang**: Methodology (equal); supervision (equal). **Chao Duan**: Resources (equal); supervision (equal). **Xiaoli Ma**: Project administration (equal); supervision (equal).

## CONFLICT OF INTEREST STATEMENT

The authors declare that they have no conflict of interest.

## ETHICS STATEMENT

This case report was approved by the Ethics Committee of Beijing Children's Hospital, Capital Medical University (IEC‐C‐006‐A04‐V.06).

## INFORMED CONSENT

Written informed consent was obtained from our patient's parents for publication of this case report, all information contained within it, and any accompanying images. Copies of the written consent are available for review by the Editor of this journal.

## Data Availability

All data generated or analyzed during this study are included in this published article.
